# Assessment of left ventricular twist by 3D ballistocardiography and seismocardiography compared with 2D STI echocardiography in a context of enhanced inotropism in healthy subjects

**DOI:** 10.1038/s41598-020-79933-4

**Published:** 2021-01-12

**Authors:** Sofia Morra, Amin Hossein, Jérémy Rabineau, Damien Gorlier, Judith Racape, Pierre-François Migeotte, Philippe van de Borne

**Affiliations:** 1 Department of Cardiovascular Diseases, Erasme Hospital, Université Libre de Bruxelles (ULB), Brussels, Belgium; 2 Laboratory of Physic and Physiology (LPHYS), Université Libre de Bruxelles (ULB), Brussels, Belgium; 3 Research Centre in Epidemiology, Biostatistics and Clinical Research, School of Public Health, Université Libre de Bruxelles (ULB), Brussels, Belgium

**Keywords:** Physiology, Cardiology

## Abstract

Ballistocardiography (BCG) and Seismocardiography (SCG) assess the vibrations produced by cardiac contraction and blood flow, respectively, by means of micro-accelerometers and micro-gyroscopes. From the BCG and SCG signals, maximal velocities (V_Max_), integral of kinetic energy (*i*K), and maximal power (P_Max_) can be computed as scalar parameters, both in linear and rotational dimensions. Standard echocardiography and 2-dimensional speckle tracking imaging echocardiography were performed on 34 healthy volunteers who were infused with increasing doses of dobutamine (5–10–20 μg/kg/min). Linear V_Max_ of BCG predicts the rates of left ventricular (LV) twisting and untwisting (both *p* < *0.0001*). The linear P_Max_ of both SCG and BCG and the linear *i*K of BCG are the best predictors of the LV ejection fraction (LVEF) (*p* < *0.0001*). This result is further confirmed by mathematical models combining the metrics from SCG and BCG signals with heart rate, in which both linear P_Max_ and *i*K strongly correlate with LVEF (R = 0.7, *p* < *0.0001*). In this setting of enhanced inotropism, the linear V_Max_ of BCG, rather than the V_Max_ of SCG, is the metric which best explains the LV twist mechanics, in particular the rates of twisting and untwisting. P_Max_ and *i*K metrics are strongly associated with the LVEF and account for 50% of the variance of the LVEF.

## Introduction

Under the assumption that the cardiovascular system equates a Newtonian system, the propulsion of blood mass into the main vessels at each cardiac contraction makes the body’s center of mass move rhythmically at each heartbeat, with a strength equal in magnitude but opposite in direction to the one of the ejected blood. Contraction of myocardial muscle, opening and closure of heart valves, blood flowing into cardiac chambers generate micro movements that are transmitted to the chest surface as low-frequency vibratory phenomena. Seismocardiography (SCG) and ballistocardiography (BCG) record the micro-vibrations generated rhythmically as a consequence of the movements of cardiac mass and blood in the major vessels, respectively, with micro-accelerometers and gyroscopes placed on the body surface^1–5^. Modern BCG and SCG can measure linear and rotational velocities and accelerations of blood stream and cardiac mass using linear and rotational channels, respectively, and in three cardinal axes using three-axial sensors (*x*: latero-lateral axis; *y*: caudo-cranial axis; *z*: antero-posterior axis)^2,6^. In such a way, a multi-dimensional assessment of blood flow and cardiac function can be obtained with 12 degrees-of-freedom (DOF). Additionally, from the BCG and SCG waveforms, linear and rotational kinetic energy (K), its temporal integral (*i*K), maximal power (P_Max_), displacements (D) and maximal velocities (V_Max_) can be computed for each contractile cycle using specific algorithms based on Newtonian equation^2^.

A growing number of evidences provide the signals recorded with BCG and SCG as good indicators of myocardial function and dysfunction. Metrics of *i*K and P_Max_ secured from BCG and SCG signals are well correlated to stroke volume (SV) and cardiac output (CO)^2^; the peak of maximum energy well represents myocardial contractility expressed as dP/dt_max_ in animal models^7^; BCG and SCG signals provide information on myocardial dysfunction after an acute coronary syndrome^8,9^ and can assess the clinical status of patients with heart failure^10^.

Left ventricular (LV) twist is the rotational movement of the LV along its longitudinal axis and results from the net difference between the torsional angles of the apical and basal rotations^11–13^. LV twist can robustly estimate myocardial function, expressed as LVEF^14–16^ in some studies or dP/dt_max_ in others^17–20^. LV untwisting rate provides the atrio-ventricular pressure gradients which assist cardiac chambers to fill during the early phase of relaxation^21,22^.

Recently, cardiac rotations have been evaluated using gyroscopes in animal models and results seem promising on the feasibility of measuring LV twist with this technique^23^.

Whether velocity and acceleration signals recorded with modern BCG and SCG can reliably estimate cardiac rotations in humans is not known.

The aims of the present study were: (1) to examine the relationships between LV twist mechanics with displacements and velocities metrics computed from the BCG and SCG signals; (2) to estimate myocardial function expressed as LVEF through the metrics of *i*K and P_Max_ obtained from the BCG and SCG signals.

## Methods

### Study protocol

The present study is based on a previous randomized, double-blind, placebo-control, cross-over study in 34 healthy volunteers aged between 18 and 50 years (code *NCT03107352* on ClinicalTrials.gov). The protocol has been approved by the local Ethics Committee and subjects gave written informed consent before being enrolled. Each subject received increasing doses of dobutamine (5–10–20 µg/kg/min) or normal saline as placebo (5–10–20 µg/kg/min). Allocation to the group receiving dobutamine first (group 1) or placebo first (group 2) was randomly determined. For each session, echocardiographic study was performed before any drug infusion (referred to as baseline) and during infusion of 5–10–20 µg/kg/min of dobutamine and placebo. According to the aims previously listed, only the dobutamine arm was considered in the present study for analysis and interpretation of results. Because of obvious changes in cardiac echocardiography following dobutamine infusion, investigators were not blinded for data analysis. Details about the protocol can be found in our previous work^2^.

Each echocardiographic study was followed by a 90-s record of linear acceleration and angular velocity signals with BCG and SCG on the skin surface.

### Echocardiographic study

Standard echocardiography was performed with the GE VIVID E95 by an expert cardiologist in non-invasive imaging. Two dimensional images of the LV were obtained from the apical 2-, 3- and 4- chamber views along the parasternal long-axis. LV volumes and LVEF were calculated by Simpson biplane methods. Stroke volume (SV), cardiac output (CO), left ventricle ejection fraction (LVEF), LV outflow tract velocity (LVOT V_max_) and its temporal integral (LVOT VTI) were measured^24,25^.

For the 2D-STI speckle tracking study, parasternal basal and apical short axis views were obtained for the measurement of basal and apical rotations. The basal level was defined from the tip of mitral valve, while the apical level was defined as the level of LV cavity without papillary muscles^26^. Three consecutive beats were recorded in a cine-loop image and analyses were performed with a dedicated software (EchoPAC version 20.1, GE Healthcare). The LV endocardial border was manually traced at the basal and apical levels and the speckle tracking thickness of LV wall was automatically selected, as previously described^15^. The width of the selected region was accommodated to the LV wall as needed.

By convention, counterclockwise rotations are expressed with positive values and clockwise rotations with negative values, looking from the apex^13^.

LV twist is defined as the net difference of angular displacements between the basal and apical rotations along the LV longitudinal axis^13^.

For the analysis of apical and basal rotation/rotational rates, LV twist and LV twisting/untwisting rates, short-axis views were considered. Data plots were then exported to Excel version 16 (Microsoft) and the peak of LV twist as well as angular rates were calculated.

The highest positive peak of the LV strain curve has been considered as the maximal angular displacement occurring during systole and is expressed in °. Rotational rates have been obtained as the temporal derivation of LV twist according to the following equation:

$$\omega = \frac{d\theta }{{dt}}$$

where ω is the angular rate and θ the angular displacement.

Rates are expressed in °/s. The positive peak of the rate of LV twist is termed “LV twisting rate”, the negative peak “LV untwisting rate”.

**Figure 1:**
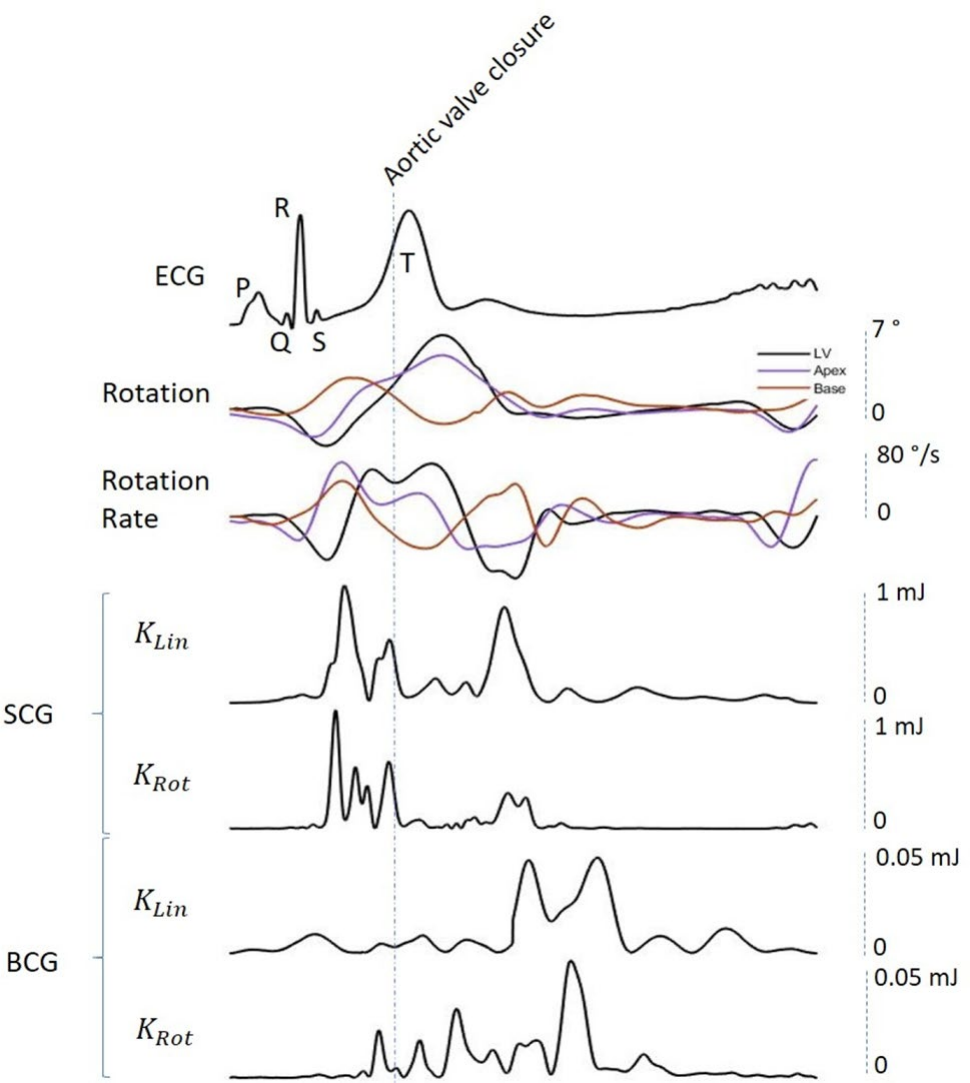
Synchronization of SCG and BCG signals with LV twist. From top to bottom: (**a**) ECG with the P-Q-R-S-T waves labeled; (**b**) LV twist, apical and basal rotations; (**c**) angular rates of LV twist, apical and basal twist rotations; (**d**) K_Lin_ SCG; (**e**) K_Rot_ SCG; (**e**) K_Lin_ BCG; (**f**) K_Rot_ BCG. The first of the two main peaks of K_Lin_ SCG (**d**) and K_Rot_ SCG (**e**) occurs before the aortic valve closure (AVC), during the ejection phase of cardiac cycle and concomitantly with the acceleration of LV twist; the second one occurs after the AVC, synchronously with the deceleration phase of LV twist. With regards to the BCG, waves of K_Lin_ and K_Rot_ occur almost exclusively after the AVC, during the diastolic phase of cardiac cycle. The Aortic Valve Closure (AVC) is labeled on the figure. *K*_*Lin*_ kinetic energy in the linear dimension, *K*_*Rot*_ kinetic energy in the rotational dimension, *SCG* seismocardiography, *BCG* ballistocardiography, *ECG* electrocardiogram, *LV* left ventricle.

Figure 1 shows the parameters of LV twist mechanics synchronized with SCG and BCG signals according to the ECG.

### SCG and BCG signal acquisition

A wearable device with two modules, one for the BCG acquisition and one for the SCG acquisition, was used. Each module contains a microelectromechanical systems (MEMS) accelerometer and gyroscope sensor (LSM6DSL, STMICROELECTRONICS) and is attached to the body with standard sticky gel electrodes. The acceleration and angular rates of the sensor were set to ± 2 g and ± 250 dps, respectively with a resolution of 0.061 mg/LSB and 4.375 mdps/LSB and an RMS noise of 80 μg/√Hz and 4 mdps/√Hz with an output bandwidth of 416 Hz. The device is controlled with a smartphone or a tablet connected via Bluetooth and collects a one-lead ECG at 200 Hz (ADS1292R, ADINSTRUMENTS) together with 3-DOF linear (LIN) accelerations and 3-DOF rotational (ROT) angular velocities from both the BCG module and the SCG module. In brief, a total of 12-DOF linear acceleration and angular velocity signals are recorded at 50 Hz. A 25 Hz hardware low-pass filter is applied.

The standard nomenclature was used: for BCG signals, *x* is the lateral (left-to-right) axis, *y* is the longitudinal (caudocranial) axis and *z* is the anteroposterior (ventrodorsal) axis; for SCG signals, the *z*-axis points in the opposite direction (dorsoventral) and the *x*-axis right-to-left.

### SCG and BCG signaling processing

The BCG module was placed in the lumbar lordosis curve, between the second and the third lumbar vertebrae, close to the subject’s center of mass. The SCG module was placed on the manubrium of the sternum below the clavicle where the great vessels emerge from the heart.

A 90-s record was acquired after each echocardiographic study using a tablet remotely connected to the device via Bluetooth and data were sent to the main server for further processing. Data were subsequently exported and processed offline using a specific toolbox written in MATLAB (version 9.5, R2018b). Further details describing this methodology can be found in previous publications^2,6,27,28^. Linear kinetic energy (K_*Lin*_) and rotational kinetic energy (K_*Rot*_) of BCG and SCG are shown on Fig. 1.

The processed signals were used to compute the integral of kinetic energy (*i*K) and the maximal power (P_Max_) for both linear and rotational channels (${i\mathrm{K}}_{\mathrm{SCG}}^{\mathrm{Lin}/\mathrm{Rot}}$; ${i\mathrm{K}}_{\mathrm{BCG}}^{\mathrm{Lin}/\mathrm{Rot}}$; ${\mathrm{P}}_{\mathrm{Max }SCG}^{\mathrm{Lin}/\mathrm{Rot}}$; ${\mathrm{P}}_{Max BCG}^{\mathrm{Lin}/\mathrm{Rot}}$). Computation of these scalar parameters has been described previously^2^ and are explained hereafter.

Knowing the acceleration of an object with a given mass m, the vector force ($\overrightarrow{F}$), the kinetic energy (K) and the power (P) can be calculated according to Eqs. (1) to (3) for the linear components and Eqs. (4) to (6) for the rotational components.

1 $$\vec{F}(t) = {\text{m}}\vec{a}(t)$$

2 $${\text{K}}_{Lin} \left( {\text{t}} \right) \, = \frac{1}{2} \cdot {\text{m}}\left( {v_{x}^{2} (t) + v_{y}^{2} (t) + v_{z}^{2} (t)} \right)$$

3 $${\text{P}}_{Lin} \left( {\text{t}} \right) \, = \vec{F}(t) \cdot \vec{v}(t)$$

where m is the mass of the object, a human being in the case of the BCG and SCG application, K_*Lin*_ is the linear kinetic energy, ${v}_{x}$, ${v}_{y}$, ${v}_{z}$ are components of the measured velocity vector $\overrightarrow{v}$ , $\overrightarrow{F}$ is the force vector and P_*Lin*_ is the linear power.

For the rotational components, the scalar metrics are calculated according to Eqs. (4) to (6).

4 $$\vec{\tau }(t) = I \cdot \vec{\alpha }(t)$$

5 $$K_{Rot} \left( t \right) = \frac{1}{2}(I_{xx} \omega_{x}^{2} \left( t \right) + I_{yy} \omega_{y}^{2} \left( t \right) + I_{zz} \omega_{zz}^{2} \left( t \right))$$

6 $${\text{P}}_{Rot} \left( {\text{t}} \right) = \,\vec{\tau }(t) \cdot \omega (t)$$

where $\overrightarrow{\tau }$ is the torque of force, I is the momentum of inertia of the object and $\overrightarrow{\alpha }$ is the angular acceleration, K_*Rot*_ is the rotational kinetic energy, ${I}_{xx}$, ${I}_{yy}$, ${I}_{zz}$ are the orthogonal components of the momentum of inertia I of the object, ${\omega }_{x}$,${\omega }_{y},$ and ${\omega }_{z}$ are components of the measured angular velocity $\overrightarrow{\omega }$, P_*Rot*_ is the rotational power.

The time integral of K_*Lin*_ and K_*Rot*_ over the cardiac cycle (CC) was computed for both SCG and BCG as in Eqs. (7) and (8).

7 $$iK_{Lin} = \mathop \smallint \limits_{CC}^{{}} K_{Lin} (t)dt$$

8 $$iK_{Rot} = \mathop \smallint \limits_{CC}^{{}} K_{Rot} (t)dt$$

Regarding the power metric, the maximal absolute value is taken to generate P_Max_ for both SCG and BCG according to Eqs. (9) and (10).

9 $$P_{{{\text{Max}}\_Lin}} = \max_{CC} \left( {P_{Lin} \left( t \right)} \right)$$

10 $$P_{{{\text{Max}}\_Rot}} = \max_{CC} \left( {P_{Rot} \left( t \right)} \right)$$

The metrics of interest described so far are computed using a specific toolbox written in MATLAB (version 9.5 R2018b, MATHWORKS).

From the BCG and SCG accelerations signals, linear and angular velocities, as well as linear and angular displacements were computed by integration and twofold integration, respectively. Velocities and displacements signals were therefore obtained for each degree of freedom in the orthogonal axis (*x*, *y*, *z*). From these, the maximum of velocity (V_Max_) and the maximum of displacement (D_Max_) were computed (Eqs. 11 and 12 respectively):

11 $$V_{{{\text{Ma}}x}} = {\text{ max}}\left( {\sqrt {\left( {v_{x}^{2} + v_{y}^{2} + v_{z}^{2} } \right)} } \right)$$

12 $$D_{Max} = \max \left( {\sqrt {(x^{2} + y^{2} + z^{2} )} } \right)$$

where ${v}_{x}$, ${v}_{y}$, ${v}_{z}$, are components of the velocity vector $\overrightarrow{v}$ ; *x*, *y*, *z* are the components of the position vector $\overrightarrow{OP}$. This was done for both linear and angular channels. D_Max_, V_Max,_ *i*K, and P_Max_ were normalized for the body surface area (BSA) of the subject.

### Statistical analysis

Statistical analysis was performed using SPSS IBM version 22 (SPSS Inc. Chicago, IL) on Windows. GRAPHPAD PRISM version 5.01 and MATLAB (MATHWORKS INC) were used for graphing figures on Windows.

Normality of data distribution was assessed using the Kolmogorov–Smirnov test.

One-way parametric or non-parametric ANOVA were applied according to data distribution.

The Spearman’s rank correlation was used to assess association between variables. Correlation coefficients range from − 1 to + 1 where 0 indicates no monotonic association and a value of 1 a perfect monotonically decreasing (− 1) or increasing (+ 1) relationship.

Generalized linear model was used to predict LV twisting and untwisting rates from velocity metrics and to predict the LVEF from metrics of P_Max_ and *i*K.

*P*-values < 0.05 were considered statistically significant.

Intra-observer variability was examined in 35 randomly selected echocardiographic images and expressed as the intraclass correlation coefficient (ICC) between the measurements of the two readings as well as their mean (± SD) difference.

## Results

Baseline characteristics, echocardiographic parameters and SCG/BCG parameters of the entire study population are presented in Table 1. The linear accelerations and angular velocities of both chest and lower-back sensors were computed to get the metrics D_Max_, V_Max_, *i*K and P_Max_. 47.1% of the participants were men, the mean age was 25 years (± 1.72), the mean BMI was 22.48 kg/m^2^ (± 2.07), while the mean SV, CO, and LVEF at baseline were respectively 62.78 ml (± 13.79), 4.32 l/min (± 0.1) and 63.3% (± 4.5).

**Table 1:** Modifications of metrics of echocardiography and SCG/BCG according to the dose of dobutamine infusion.

Parameters	Baseline	5 µg/kg/min	10 µg/kg/min	20 µg/kg/min	*P*_*All*_
Sex (% male)	47.1	–	–	–	
Age	25 ± 1.7	–	–	–	
BMI	22.5 ± 2.1	–	–	–	
SV	61.2 ± 12.0	71.8 ± 13.0	82.5 ± 19.0	84.7 ± 18.0	*0.0001*
CO	4.1 ± 0.8	4.7 ± 0.8	6.4 ± 1.0	8.8 ± 1.9	*0.0001*
LVOT V_max_	20.2 ± 2.6	24.3 ± 3.3	27.7 ± 2.8	28.1 ± 3.3	*0.0001*
LVOT VTI	0.98 ± 0.13	1.26 ± 0.24	1.72 ± 0.22	1.85 ± 0.25	*0.0001*
HR	70.6 ± 10.3	72.4 ± 10.4	87.3 ± 16.5	115.2 ± 21.6	*0.0001*
LVEF	63.3 ± 4.5	69.2 ± 8.5	78.9 ± 4.7	81.7 ± 4.0	*0.0001*
**SCG**					
${D}_{Max}^{Lin}$	0.15 ± 0.08	0.22 ± 0.10	0.23 ± 0.08	0.19 ± 0.08	*0.003*
${\mathrm{D}}_{\mathrm{max}}^{\mathrm{Rot}}$	11.7 ± 8.6	21.1 ± 16.5	24.9 ± 12.2	23.3 ± 11.9	*0.0001*
${V}_{Max}^{Lin}$	1.69 ± 0.7	2.87 ± 1.13	3.54 ± 1.14	3.62 ± 1.30	*0.0001*
${V}_{Max}^{Rot}$	244.0 ± 150.9	527.5 ± 356.8	746.2 ± 426.2	816.6 ± 512.0	*0.0001*
${\mathrm{iK}}_{\mathrm{Lin}}$	40.0 ± 30.0	80.0 ± 50.0	120.0 ± 60.0	110.0 ± 70.0	*0.0001*
${\mathrm{iK}}_{\mathrm{Rot}}$	20.0 ± 20.0	60.0 ± 60.0	90.0 ± 80.0	100.0 ± 90.0	*0.0001*
${P}_{Max}^{Lin}$	11.5 ± 1.4	13.8 ± 1.8	15.4 ± 1.6	15.8 ± 1.9	*0.0001*
${P}_{Max}^{Rot}$	0.009 ± 0.009	0.08 ± 0.05	0.47 ± 0.45	0.18 ± 0.06	*0.0001*
**BCG**					
${D}_{Max}^{Lin}$	0.05 ± 0.02	0.06 ± 0.02	0.06 ± 0.02	0.07 ± 0.03	*0.002*
${D}_{Max}^{Rot}$	2.1 ± 1.1	2.9 ± 1.1	3.6 ± 1.3	4.3 ± 1.6	*0.0001*
${V}_{Max}^{Lin}$	0.5 ± 0.2	0.7 ± 0.2	0.8 ± 0.3	1.0 ± 0.3	*0.0001*
${V}_{Max}^{Rot}$	49.0 ± 31.7	95.5 ± 62.5	124.7 ± 82.8	147.7 ± 86.7	*0.0001*
${\mathrm{iK}}_{\mathrm{Lin}}$	6.0 ± 4.0	8.0 ± 4.0	10.0 ± 5.0	10.0 ± 7.0	*0.0001*
${\mathrm{iK}}_{\mathrm{Rot}}$	0.7 ± 0.7	2.0 ± 0.8	3.0 ± 2.0	4.0 ± 3.0	*0.0001*
${\mathrm{P}}_{\mathrm{max}}^{\mathrm{Lin}}$	0.3e^−3^ ± 0.0e^−3^	0.7e^−3^ ± 0.0002	0.7e^−3^ ± 0.4e^−3^	1.0e^−3^ ± 0.3e^−3^	*0.0001*
${\mathrm{P}}_{\mathrm{max}}^{\mathrm{Rot}}$	0.04e^−3^ ± 0.0e^−3^	0.3e^−3^ ± 0.03e^−3^	0.3e^−3^ ± 0.04e^−3^	0.7e^−3^ ± 0.09e^−3^	*0.0001*

One-way parametric or non-parametric ANOVA was applied according to data distribution. For clarity purpose, continuous variables are all presented as mean ± SD.

Statistical significance was set at 0.05 and emphasized in italics.

*BMI* body mass index (kg/m^2^), *SV* stroke volume (ml), *CO* cardiac output (l/min), *LVOT V*_*max*_ left ventricle outflow tract maximal velocity (m/s), *LVOT VTI* left ventricle outflow tract integral of velocity, *HR* heart rate (bmp); *LVEF* left ventricle ejection fraction (%); ${D}_{max}^{Lin}$ maximal displacement in the linear dimension (mm/m^2^), ${D}_{Max}^{Rot}$ maximal displacement in the rotational dimension (°/m^2^), ${V}_{max}^{Lin}$ maximal velocity in the linear dimension (mm/s/m^2^), ${V}_{max}^{Rot}$ maximal velocity in the rotational dimension (°/s/m^2^), ${iK}_{Lin}$ integral of kinetic energy in the linear dimension (µJ s/m^2^), ${iK}_{Rot}$ integral of kinetic energy in the rotational dimension (µJ s/m^2^), ${P}_{Max}^{Lin}$ maximal power in the linear dimension (J/s/m^2^), ${P}_{Max}^{Rot}$ maximal power in the rotational dimension (mJ/s/m^2^).

Maximum of linear displacements and angular displacements computed with SCG and BCG have been associated with the LV twist. Results are shown in Table 2.

**Table 2:** Associations of displacements from SCG and BCG with the peak of LV twist from 2D STI echocardiography.

	SCG		BCG	
	${\mathrm{D}}_{\mathrm{Max}}^{\mathrm{Lin}}$	${\mathrm{D}}_{\mathrm{max}}^{\mathrm{Rot }}$	${\mathrm{D}}_{\mathrm{max}}^{\mathrm{Lin}}$	${\mathrm{D}}_{\mathrm{max}}^{\mathrm{Rot }}$
LV twist	R = 0.05; p = 0.57	R = 0.17; *p* = *0.05*	R = 0.18; *p* = *0.04*	R = 0.3; *p* = *0.001*

Spearman's correlation of velocities computed from the SCG and BCG signals in both linear and rotational channels with peak of LV twist obtained with 2D STI echocardiography.

${D}_{max}^{Lin}$ maximal displacement in the linear dimension (mm/m^2^), ${D}_{Max}^{Rot}$ maximal displacements in the rotational dimension (°/m^2^), *BCG* ballistocardiograph, *SCG* seismocardiography.

LV twist poorly correlated with displacements obtained from SCG and BCG signals. Specifically, it correlates with D_Max_ from BCG signal both in the linear and in the rotational dimension (R = 0.18, *p* = *0.04*; R = 0.30, *p* = *0.001*, respectively) and with D_Max_ from the SCG signal in the rotational dimension only (R = 0.17, *p* = *0.05*), but the strength of association is very week.

Maximum of velocities computed with SCG and BCG have been associated with rates of LV twisting and untwisting. Results are shown in Table 3.

**Table 3:** Associations of velocities from SCG and BCG with LV twisting and untwisting rates from 2D STI echocardiography.

	SCG				BCG			
	${\mathrm{V}}_{\mathrm{max}}^{\mathrm{Lin}}$	${\mathrm{V}}_{\mathrm{max}}^{\mathrm{Rot}}$	*i*K_*Lin*_	*i*K_*Rot*_	${\mathrm{V}}_{\mathrm{max}}^{\mathrm{Lin}}$	${\mathrm{V}}_{\mathrm{max}}^{\mathrm{Rot}}$	*i*K_*Lin*_	*i*K_*Rot*_
LV twisting rate	R = 0.41; *p* < *0.0001*	R = 0.37; *p* = *0.002*	R = 0.27; *p* = *0.002*	R = 0.34; *p* < *0.0001*	R = 0.50; *p* < *0.0001*	R = 0.45; *p* < *0.0001*	R = 0.37; *p* < *0.0001*	R = 0.46; *p* < *0.0001*
LV untwisting rate	R = − 0.35; *p* < *0.0001*	R = − 0.36; *p* < *0.0001*	R = − 0.23;* p* = *0.009*	R = − 0.34; *p* < *0.0001*	R = − 0.45; *p* < *0.0001*	R = − 0.47; *p* < *0.0001*	R = − 0.34; *p* < *0.0001*	R = − 0.46; *p* < *0.0001*

Spearman's correlation of velocities and *i*K computed from the SCG and BCG signals in both linear and rotational channels with LV twisting and untwisting rates obtained from 2D STI echocardiography.

Statistical significance was set at 0.05 and emphasized in italics.

${V}_{Max}^{Lin}$ maximal velocity in the linear channel (mm/s/m^2^), ${V}_{Max}^{Rot}$ maximal velocity in the rotational channel (°/s/m^2^), *i*K_*Lin*_ integral of kinetic energy in the linear channel (µJ s/m^2^), *i*K_*Rot*_ integral of kinetic energy in the rotational channel (µJ s/m^2^).

LV twisting rate is related to V_Max_ of the SCG (R = 0.41, *p* < *0.0001*; R = 0.37, *p* < *0.0001* for the linear and rotational dimensions, respectively) and to V_Max_ of the BCG (R = 0.50, *p* < *0.0001*; R = 0.45, *p* < *0.0001* for the linear and rotational channels, respectively). LV untwisting rate correlates as well with V_Max_ of the SCG (R = − 0.35, *p* < *0.0001*; R = − 0.36, *p* < *0.0001*, for the linear and rotational dimensions respectively) and with V_Max_ of the BCG (R = − 0.45, *p* < *0.0001*; R = − 0.47, *p* < *0.0001*, for the linear and the rotational dimensions respectively).

The generalized linear model, where linear and rotational velocities of both SCG and BCG were taken into the model to predict the LV twisting and untwisting rates, shows that the linear V_Max_ of the BCG is the best predictor of the LV twisting and untwisting rates, rather than the V_Max_ of the SCG (Table 4).

**Table 4:** Estimation of LV twisting rate and untwisting rate through metrics secured from the SCG and BCG signals.

	Coefficient of proportionality	Confidence interval 95%	*p*-value
**Estimation of LV twisting rate**			
BCG linear V_Max_	78.5	38.4; 118.5	*0.0001*
SCG linear V_Max_	7.3	− 4.27; 18.9	0.22
BCG rotational V_Max_	0.0081	− 0.0075; 0.24	0.31
SCG rotational V_Max_	0.0011	− 0.0023; 0.046	0.53
**Estimation of LV untwisting rate**			
BCG linear V_Max_	− 72.2	− 113.8; − 30.6	*0.0001*
SCG linear V_Max_	1.8	− 9.87; 13.49	0.76
BCG rotational V_Max_	− 0.16	− 0.33; 0.01	0.06
SCG rotational V_Max_	− 0.033	− 0.069; 0.003	0.08

Generalized linear model. Variables taken into the model were: SCG linear V_Max_, BCG linear V_Max_, SCG rotational V_Max_, BCG rotational V_Max_. Outcomes: LV twisting rate (°/s) and LV twisting rate (°/s).

*V*_*max*_ maximal velocity (mm/s/m^2^), *BCG* ballistocardiography, *SCG* seismocardiography, *LV* left ventricle.

For one unit increase of V_Max_ of the BCG, the rate of LV twisting hastens of 78.5 units and the rate of LV untwisting accelerates of − 72.2 units. Linear V_Max_ of SCG and rotational V_Max_ of BCG and SCG do not estimate rates of LV twisting and untwisting.

Correlations of parameters of cardiac contractile function with *i*K and P_Max_ derived from SCG and BCG waveforms have been calculated and are summarized in Table 5.

**Table 5:** Associations of P_max_ and *i*K computed from SCG and BCG signals with hemodynamic parameters obtained with standard echocardiography.

Parameter	LVEF	LVOT V_max_	LVOT VTI
**SCG**			
${\mathbf{P}}_{\mathbf{M}\mathbf{a}\mathbf{x}}^{\mathbf{L}\mathbf{i}\mathbf{n}}$	R = 0.51, *p* < *0.0001*	R = 0.55, *p* < *0.0001*	R = 0.46, *p* < *0.0001*
${\mathbf{P}}_{\mathbf{M}\mathbf{a}\mathbf{x}}^{\mathbf{R}\mathbf{o}\mathbf{t}}$	R = 0.52, *p* < *0.0001*	R = 0.49, *p* < *0.0001*	R = 0.43, *p* < *0.0001*
${\mathbf{i}\mathbf{K}}_{\mathbf{L}\mathbf{i}\mathbf{n}}$	R = 0.36, *p* < *0.0001*	R = 0.39, *p* < *0.0001*	R = 0.38, *p* < *0.0001*
${\mathbf{i}\mathbf{K}}_{\mathbf{R}\mathbf{o}\mathbf{t}}$	R = 0.51, *p* < *0.0001*	R = 0.47, *p* < *0.0001*	R = 0.42, *p* < *0.0001*
**BCG**			
${\mathbf{P}}_{\mathbf{M}\mathbf{a}\mathbf{x}}^{\mathbf{L}\mathbf{i}\mathbf{n}}$	R = 0.50, *p* < *0.0001*	R = 0.66, *p* < *0.0001*	R = 0.49, *p* < *0.0001*
${\mathbf{P}}_{\mathbf{M}\mathbf{a}\mathbf{x}}^{\mathbf{R}\mathbf{o}\mathbf{t}}$	R = 0.46, *p* < *0.0001*	R = 0.60, *p* < *0.0001*	R = 0.52, *p* < *0.0001*
${\mathbf{i}\mathbf{K}}_{\mathbf{L}\mathbf{i}\mathbf{n}}$	R = 0.36, p < 0.0001	R = 0.51, p < 0.0001	R = 0.40, *p* < *0.0001*
${\mathbf{i}\mathbf{K}}_{\mathbf{R}\mathbf{o}\mathbf{t}}$	R = 0.45, *p* < *0.0001*	R = 0.62, *p* < *0.0001*	R = 0.54, *p* < *0.0001*

Spearman's correlations between *i*K and P_Max_ computed from the BCG and SCG in linear and rotational channels and LVEF, LVOT V_max_, LVOT VTI for all levels of dobutamine administration.

*SCG* seismocardiography, *BCG* ballistocardiography, *iK* maximal kinetic energy, *P*_*Max*_ maximal Power, *LVEF* left ventricle ejection fraction, *LVOT V*_*max*_ left ventricle outflow tract maximal velocity, *LVOTI VTI* left ventricle outflow tract velocity time integral.

LVEF is associated with all the parameters derived from the BCG and SCG. The strongest association is observed with the metrics derived from the SCG, specifically *i*K_*Rot*_ (R = 0.51), ${\mathrm{P}}_{\mathrm{max}}^{\mathrm{Rot}}$ (R = 0.52) and ${\mathrm{P}}_{\mathrm{max}}^{\mathrm{Lin}}$ (R = 0.51). When the linear channel for *i*K is considered as the comparative factor, the strength of association is lost to some extent, both for the SCG and the BCG (R = 0.36, *p* < *0.0001*; R = 0.36, *p* < *0.0001,* respectively).

The association is slightly weaker when the same metrics are computed from the rotational channel of the BCG (R = 0.45 for *i*K_*Rot*_; R = 0.46 for ${\mathrm{P}}_{\mathrm{Max}}^{\mathrm{Rot}}$). Variables computed from the BCG and the SCG signals that best predict the LVEF are shown in Table 6.

**Table 6:** Estimation of LVEF.

	Coefficient	Confidence interval 95%	*p-*value
BCG linear P_Max_	0.12	0.05; 0.19	*0.0001*
SCG linear P_Max_	1.30	0.03; 2.6	*0.04*
BCG linear *i*K	4.35	1.33; 7.37	*0.005*

Generalized linear model. Variables taken into the model were SCG linear P_Max_, BCG linear P_Max_, SCG rotational P_Max_, BCG rotational P_Max_, SCG linear *i*K, BCG linear *i*K, SCG rotational *i*K, BCG rotational *i*K. Outcome: LVEF.

*P*_*Max*_ maximal power, *iK* integral of kinetic energy, *BCG* ballistocardiography, *SCG* seismocardiography, *LVEF* left ventricle ejection fraction.

The linear P_Max_ of both BCG and SCG as well as the linear *i*K of the BCG are the variables that best estimate the LVEF. In particular, the LVEF increases by 0.12% for one unit increase of the linear P_Max_ of the BCG (J/s/m^2^), by 1.3% for one unit increase of the linear P_Max_ of the SCG (J/s/m^2^), by 4.35% for one unit increase of the linear *i*K of the BCG (µJ s/m^2^).

**Figure 2:**
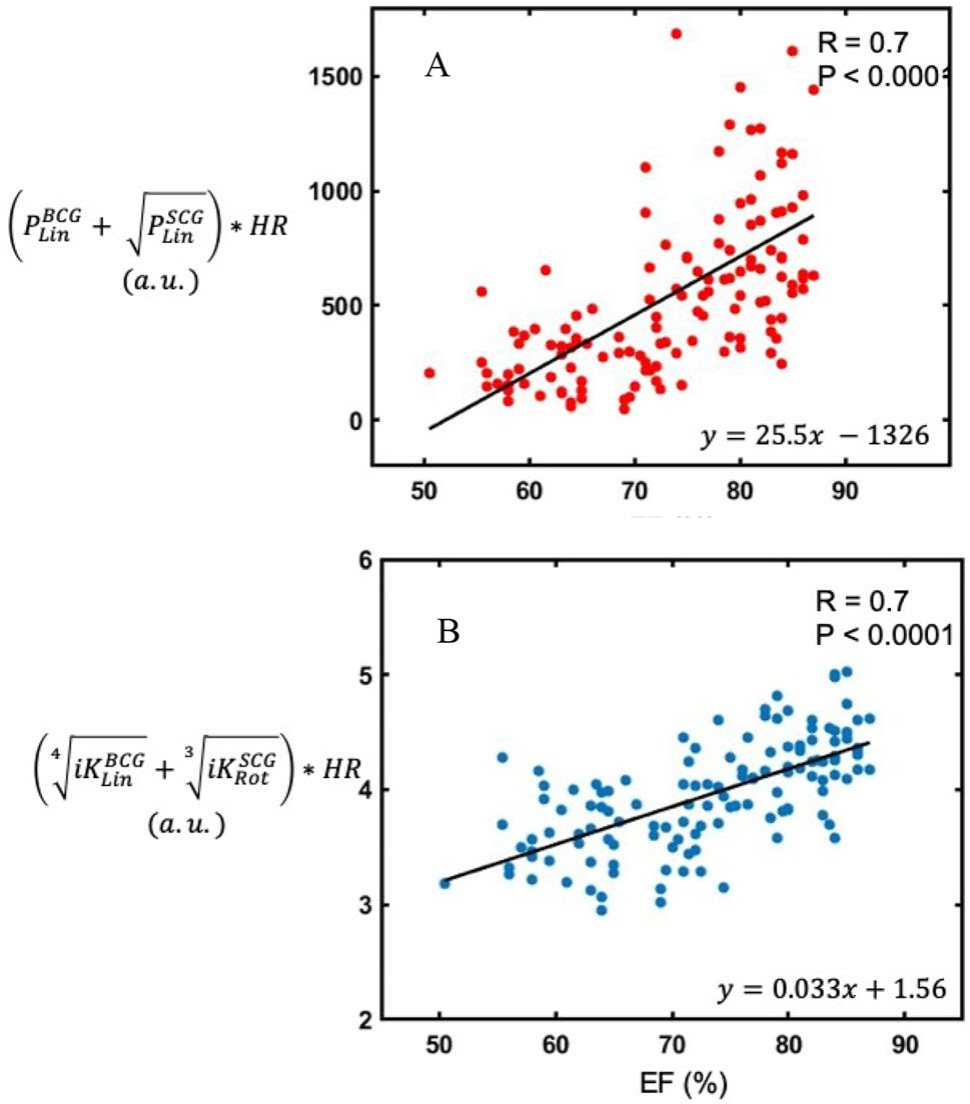
Correlations between metrics of BCG and SCG with the LVEF. Mathematical models combining: (**A**) linear P_Max_ of SCG and BCG with the LVEF and (**B**) *i*K of SCG and BCG with the LVEF (both R = 0.7, p < 0.0001).

By empirically combining the metrics computed from the BCG and SCG signals with the HR, two mathematical models have been built, in which P_Max_ and *i*K are the metric which strongest correlates with LVEF (both R = 0.7, *p* < *0.0001*), in line with the results from the generalized linear model (Fig. 2). In particular, the first model (panel A) combined the HR with ${\mathrm{P}}_{\mathrm{Lin}}^{\mathrm{BCG}}$ and $\sqrt{{\mathrm{P}}_{\mathrm{Lin}}^{\mathrm{SCG}}}$. The second model (panel B) combined the HR with $\sqrt[4]{{iK}_{Lin}^{BCG}}$ and $\sqrt[3]{{iK}_{Rot}^{SCG}}.$

## Discussion

The twisting motion of the heart makes the blood mass flow from the cardiac chambers to the main extracardiac vessels. This phenomenon can be measured with 2D STI echocardiography and rates of LV twisting and untwisting can be derived^13,29–31^.

From this study, we have observed that, in a context of incremental rise of myocardial inotropism in healthy subjects:

1. LV twisting and untwisting rates are well associated with the velocity signals measured with the BCG and SCG; velocity signals of BCG are best indicators of the twisting motion of the LV than those of the SCG. Indeed, among the velocity variables obtained from the BCG and SCG signals, the velocity vector of the BCG in the linear dimension, rather than the one of the SCG, is the one which best estimates the modifications of LV twisting and untwisting rates;
2. Signals of kinetic energy and power computed from the BCG and the SCG signals provide information on the contractility status of the heart and significantly predict changes in LVEF. In particular, the linear *i*K of the BCG is the metric that best predicts the changes of LVEF. When metrics of P_Max_ and *i*K are combined with the HR, they are both related to the LVEF with a strength of 0.70.

Previously to our observations, Marcelli et al. proposed gyroscopic sensors as a feasible and reliable method to assess cardiac rotations in animal models^23^. In the present investigation, we confirm as well that 3-axis micro-accelerometers and gyroscopes provide valuable information on the twisting motion of the heart, specifically on rates of twisting and untwisting. Indeed, associations have been found between velocity vectors of BCG and SCG and rates of twisting and untwisting.

In particular, changes of LV twisting and untwisting rates are significantly predicted by the linear velocity of the BCG, rather than by the same metrics of the SCG.

During a contractile cycle, blood flows inside and outside cardiac chambers, forming vortexes, which relocates blood mass across the LV according to the cycle phase^32–34^. Myocardial wall mechanics may be involved in the dynamic relocation of blood flow across cardiac chambers^35^. Indeed, during early diastole, blood fills cardiac chambers creating vortex rings directed from the LV base to the apical regions^33,35,36^, while during systole, blood flow is relocated towards the LVOT and the flow-vector is thus directed from the LV apex to the outflow tract^32,33^. In the normal heart and great vessels, in the absence of a stenotic narrowing, the blood stream is laminar, meaning that the direction and velocity of blood flow in adjacent sections are similar and uniform. This laminar flow profile is termed “plug flow”^37^. As such, blood flow velocity in the LVOT is laminar, in the absence of aortic stenosis^37^. Early during filling, diastolic vortex rings transport blood mass across cardiac chambers, contributing to about 15% of the filling volume in normal hearts^38^. The LV untwisting rate plays a pivotal role in the generation of these diastolic vortex rings^33,39,40^.

On the basis of these consolidated knowledges, authors speculate that the better estimation of rates of LV twisting and untwisting by the BCG linear velocity probably reflects the laminar profile of the blood stream in the LVOT and the vortex formation during early filling, respectively^35,38^. This latter finding is of main importance since the untwisting rate is a crucial phenomenon assisting and driving the diastolic filling and contributes to the cardiac torsional reserve^41^, which can decline in failing hearts^42^.

According to what is stated in the current literature, that is, the SCG records the vibratory phenomena produced by myocardial contraction and then transmitted to the local chest surface^43,44^, we would have expected the SCG velocities vectors to better correlate with the torsional motion of the LV. This is not exactly what we observed, since velocity vectors of SCG do not explain the behavior of LV twist mechanic in this very context of enhanced inotropism, while the linear velocity obtained from the BCG signal provide more information on the global LV twist mechanic. One possible explanation is that, since the resultant SCG signal is generated by several physiological phenomena including not only cardiac contraction, but also heart valves closure and opening, blood turbulence, momentum changes^45^, the sum of these phenomena other than cardiac contraction only may be responsible for the weaker correlation with the LV torsion observed.

In our previous work, we introduced *i*K and P_Max_ as reliable indexes of the contractile status of the heart, they can follow changes in cardiac contractility over a wide range of inotropic states and the linear *i*K of the BCG is the metric which best correlates with the SV^2^. In the current study, we provide the new evidence that the same metric is a good index also of the LVEF. In particular, we found that the linear *i*K of the BCG explains the largest changes of the LVEF in this very context of incremental rise of cardiac inotropism. The mathematical models obtained by combining metrics of P_Max_ and *i*K confirm that these metrics are strongly associated with the LVEF (both R = 0.70), thus accounting for 49% of the variance of the LVEF. While this model is of interest, it was empirically constructed and its main contribution is not to be used as it is but rather to highlight that more advanced statistical techniques, such as machine learning algorithms, could allow to build a function explaining LVEF changes on the basis of BCG and SCG linear and rotational channels.

LVOT V_max_ is the maximal velocity of blood flow across the left ventricle outflow tract, and the temporal integral of LVOT V is the integrated velocity throughout a cardiac cycle (LVOT VTI)^37^; both are important echocardiographic parameters of cardiac systolic function and cardiac output^46^. We found that all the metrics computed from the SCG and the BCG are correlated with LVOT V_max_ and LVOT VTI, but metrics of the BCG are better associated than the same metrics of the SCG. This difference between metrics of SCG and metrics of BCG can be explained through the different genesis of the signals. As explained above, while the SCG signal is the resultant of several combined cardiac phenomena, BCG signals are generated mainly by blood acceleration and deceleration into the aorta and its main branches^47^.

The results of this investigation are of main importance since they shed further light on the physiological genesis of the SCG and BCG signals, enriching the current knowledges on the SCG and BCG. Indeed, we demonstrated that the twisting motion of the heart play a fundamental role in the genesis of the BCG signal but no in the one of the SCG. The BCG signal, specifically the linear velocity, can estimate rates of LV twisting and untwisting, thus providing significant information on the twisting motion of the heart. Since the LV twist is impaired early in a variety of cardiomyopathies^48,49^, scalar parameters secured from BCG signals may prove useful in the detection of LV dysfunction in patients with cardiac diseases. Scalar metrics secured from SCG and BCG signals can also estimate the LVEF. This information complements the current knowledge on the SCG and BCG, by adding that metrics of *i*K can estimate not only the CO and SV^2^, thus providing information on the hemodynamic aspect of cardiovascular system, but can estimate as well the LVEF, thus providing information on the contractility status of the heart.

Acknowledged that metrics of kinetic energy and velocities secured by the SCG and BCG signals seem promising in the monitoring of hemodynamic profile and cardiac contractility in healthy subjects, this technique may be extended to patients suffering from cardiovascular diseases, with the promise to remotely monitor their cardiovascular conditions. Thanks to the easy-to-use properties of the device, cardiac patients might be empowered to follow their own medical conditions in a near future. Additionally, to our knowledge, markers of myocardial contractility are not provided by the smartwatches currently in use and this device may complement the existing ones by adding the cardiac *i*K as a new parameter for myocardial contractility.

### Limitations

Several possible limitations need consideration. Firstly, this is a retrospective data analysis based on a previous randomized double-blind investigation (code *NCT03107352* on ClinicalTrials.gov), enrolling exclusively healthy subjects with a small final sample size (N = 34)^2^. Additionally, results should not be applied to cardiovascular patients. In this previous study, the sample size was calculated and accounted for 32 included subjects. The sample size was estimated by assuming that cardiac contractility measured by the SCG/BCG device would increase by over 10% for the smallest dose of dobutamine, similar to its effect on SV^50^. Power analysis demonstrated that to achieve 80% power at α = 0.05, at least 32 subjects were needed. Therefore, 36 subjects were recruited to account for possible dropouts and/or technical failures. For the final analysis, 34 participants completed all the echocardiographic, ECG, and SCG/BCG acquisitions (there were 2 dropouts)^2^.

Parameters from standard and 2D STI echocardiography were measured by only one operator for logistical reasons, thus precluding the calculation of interobserver variability. To test intra-observer variability, intraclass correlation coefficient (ICC) was calculated in 35 random 2D STI echocardiographs using a two-way mixed model. The ICC was 0.94 (IC95% 0.88; 0.97) for the LV twist, 0.90 (IC95% 0.80; 0.95) for the LV twisting rate and 0.92 (IC95% 0.92 0.85; 0.96) for the LV untwisting rate. Means (± SEM) of the difference between the measurements of first and the second readings were: 0.02° ± 0.61° for the LV twist (p = 0.97); 1.44 ± 6.51°/s for the LV twisting rate (p = 0.83); 6.88 ± 7.32°/s for the LV untwisting rate (p = 0.35).

Statements about the physiological relationship between the velocity parameters of SCG and BCG and LV twisting/untwisting rates are speculative and have been postulated on the basis of the current knowledge of blood fluid dynamics. Thus, our results do not enable us to confirm such a hypothesis, which remain conjectural. Indeed, a quantification of blood flow by mean of particle image velocimetry is needed to confirm this hypothesis and may be the object of further researches.

Despite what was highlighted up to this point, authors believe that these limitations do not preclude our conclusions.

We conclude the follow: (1) in a context of pharmacologically induced incremental rise of myocardial inotropism in healthy subjects, rates of LV twisting and untwisting play a central role in the physiological genesis of the BCG signal but not of the SCG signal; (2) the linear velocity of BCG, rather than the one of the SCG, predicts the behavior of LV twisting and untwisting rates and this estimation of LV twist mechanic by the BCG signals may reflect indirectly the blood stream and relocation within cardiac chambers driven by LV twist mechanic according to the phase of a contractile cycle; (3) metrics of P_Max_ and *i*K secured from the SCG and BCG waveforms provide reliable information on the contractile status of the heart and account for 49% of the variance of the LVEF.

## Data Availability

Data are available from the authors upon reasonable request.
